# Ethnic-Specific Threshold Analysis and BMI and Waist Circumference Cutoffs for Cardiovascular Disease and Subjective Wellbeing: Results using Data from the UK Biobank

**DOI:** 10.1007/s40615-024-02193-9

**Published:** 2024-10-11

**Authors:** Mubarak Patel, Mohammed Aadil Buchya, Olalekan Uthman

**Affiliations:** Warwick Evidence, Warwick Medical School (WMS), University of Warwick, Coventry, CV47AL UK

**Keywords:** Threshold, Cutoff, Cardiovascular disease, Subjective wellbeing, Ethnicity

## Abstract

**Objectives:**

We aimed to identify ethnicity-specific BMI and waist circumference cutoffs for cardiovascular disease (CVD) and to define optimal thresholds for CVD risk and subjective wellbeing (SWB) through predictive modelling, to inform precise public health initiatives.

**Methods:**

We used data from 296,767 UK Biobank participants and adjusted logistic and linear regression models for CVD and SWB, respectively, complemented by receiver operating characteristic analysis, to explore optimal risk thresholds of CVD in six different ethnic groups and to calculate ethnicity-specific cutoffs of BMI and waist circumference (WC) to further elucidate the relationships between demographic factors and cardiovascular risk among diverse populations.

**Results:**

The logistic regression model of CVD revealed moderate discriminative ability (AUROC ~ 64–65%) across ethnicities for CVD status, with sensitivity and specificity values indicating the model’s predictive accuracy. For SWB, the model demonstrated moderate performance with an AUROC of 63%, supported by significant variables that included age, BMI, WC, physical activity, and alcohol intake. Adjusted-incidence rates of CVD revealed the evidence ethnic-specific CVD risk profiles with Whites, South Asians and Blacks demonstrating higher predicted CVD events compared to East Asians, mixed and other ethnic groups.

**Conclusion:**

Alterations of ethnicity-specific BMI and waist circumference are required to ensure ethnic minorities are provided with proper mitigation of cardiovascular risk, addressing the disparities observed in CVD prevalence and outcomes across diverse populations. This tailored approach to risk assessment can facilitate early detection, intervention and management of CVD, ultimately improving health outcomes and promoting health equity. The moderate accuracy of predictive models underscores the need for further research to identify additional variables that may enhance predictive accuracy and refine risk assessment strategies.

**Supplementary Information:**

The online version contains supplementary material available at 10.1007/s40615-024-02193-9.

## Introduction

Cardiovascular diseases (CVDs) pose a significant global health burden, contributing to a substantial portion of global morbidity and mortality [1]. Extensive research has focused on identifying modifiable risk factors for CVD development, including smoking and physical activity [2–5]. However, there is increasing recognition that a comprehensive understanding of health outcomes requires consideration of factors beyond physical health indicators and modifiable risk factors.

Despite advancements in medical treatment and preventive strategies, the burden of CVD continues to affect certain populations, with marked disparities observed across ethnic groups [6]. Ethnicity plays a critical role in shaping cardiovascular risk profiles, with evidence suggesting that individuals from minority ethnic backgrounds often experience higher rates of CVD and its associated complications compared to their white counterparts [7–9]. These disparities are influenced by a complex interplay of genetic, socioeconomic, environmental, and lifestyle factors, highlighting the need for targeted approaches to risk assessment and intervention.

In recent years, there has been a growing recognition of the importance of subjective wellbeing (SWB) as a key determinant of overall health and quality of life [10]. SWB encompasses various dimensions such as life satisfaction and has been shown to influence health outcomes, including cardiovascular health [11, 12]. Research suggests that lower SWB is associated with a higher incidence of risk factors such as hypertension [13, 14], poor cholesterol profiles, and higher inflammatory markers [15], all of which are linked to the development and progression of CVD. Furthermore, SWB may affect adherence to preventative behaviours for CVD, such as physical activity and smoking cessation, thereby influencing long-term health outcomes [16, 17]. However, research examining the relationship between demographic factors, CVD risk and SWB, particularly in within the context of ethnic diversity, remains limited.

The UK Biobank (UKB) offers a unique opportunity to address these knowledge gaps by providing access to extensive health and demographic data from a large and diverse population, including those from diverse ethnic backgrounds. Leveraging this rich dataset, our study aims to investigate the associations between demographic characteristics, CVD risk and SWB among UK Biobank participants, with a particular focus on understanding how these relationships vary across ethnic groups.

By examining demographic factors such as age, body mass index (BMI), waist circumference (WC), lifestyle behaviours and psychosocial factors, our study seeks to identify ethnic-specific risk thresholds and elucidate the mechanisms underlying disparities in CVD risk and SWB. The findings from this research have the potential to inform targeted interventions and public health strategies aimed at reducing health inequities and improving cardiovascular outcomes among diverse populations. Moreover, this study contributes to the broader literature on cardiovascular health and wellbeing, advancing our understanding of the complex interplay between demographic factors, health outcomes and quality of life.

## Methods

### Data Source

Data were obtained from the UK Biobank [18], a large prospective study of around half a million participants in the UK who were enrolled at ages from 40 to 69 years between 2006 and 2010. The study, designed to explore the intricate relationship between genetics, environmental factors and health outcomes, collected data under informed consent and with ethical approval.

In this cross-sectional study of Biobank data, pertinent data were related to CVD and SWB. This cohort contained 296,767 participants with a valid SWB score as part of UK Biobank application number 103665. Beyond wellbeing, the present study utilised data on demographic characteristics, health-related outcomes, mental health, general wellbeing and physical activity variables. As ethnicity was a key factor in the analysis, there were no restrictions on the ethnicity of participants. Researchers can access the data through an established application process, ensuring transparency and openness. Quality control measures are in place to maintain the reliability and validity of the extensive dataset.

### Key Variables

#### Cardiovascular Disease Types

In the UKB, the participants were queried about various cardiovascular events such as myocardial infarction or stroke, which represent distinct cardiovascular subtypes. Participants provided the dates of these events if applicable. To facilitate analysis, binary variables were created to indicate the presence or absence of any CVD event. Participants who reported experiencing any cardiovascular event were assigned a value of ‘1’, while those who reported no such events were assigned a value of ‘0’. Additionally, binary variables were created to capture the occurrence of specific cardiovascular subtypes as defined by the International Statistical Classification of Diseases and Related Health Problems, 10th Revision (ICD-10) [19]. These subtypes included acute rheumatic fever, chronic rheumatic heart disease, hypertensive diseases, ischaemic heart diseases, pulmonary heart diseases, other forms of heart disease, cerebrovascular diseases, diseases of arteries and capillaries, diseases of veins and lymphatic vessels not elsewhere classified and unspecified disorders of the circulatory system. Each subtype was represented by a separate binary variable, enabling the investigation of specific disease entities within the broader spectrum of cardiovascular health.

#### Ethnicity

The ethnicities of UKB participants were self-reported and recorded in six categories: White, Mixed, South Asian, Black, East Asian and other. Participants with mixed ethnicity were those who belonged to more than one ethnic group, while participants categorised as other were of an ethnicity outside of the other available choices. This categorization aims to encompass diverse ethnic backgrounds and is crucial for addressing research objectives related to ethnicity’s role in moderating the relationship between ethnicity, CVD and SWB. These categorisations are broadly similar to the ones used in the UK census [20].

#### Subjective Wellbeing Measures

SWB in the UKB was assessed using various measures. The study aggregated score form five domains: general happiness and satisfaction with family, friendship, health, and financial situation, resulting in a continuous variable ranging from 0 to 100. Higher scores indicated higher levels of SWB, serving as the primary outcome measure. This approach aligns with previous studies [21–23] due to the absence of a single wellbeing measure in the UKB. Statistical analyses were conducted to validate the combination of these variables into a composite score.

### Statistical Analysis

Demographic characteristics were chosen based on variables that have been found to be associated with CVD or SWB [2, 24–26]. This includes age, body mass index (BMI), waist circumference (WC), ethnic group, sex, current smoking status, depression, alcohol intake, how many days a week one performs moderately intense physical activity and handgrip strength. Current smoking status categorises participants into never smoked, ex-smoker or current smoker. Depression is a binary variable where participants were asked if they experienced depression in the past 6 months. Alcohol intake was assessed via self-reported frequency of consumption, such as only on never, special occasions, 1–3 times a months, 1–2 times a week and 3–4 times a week or daily. The normality of continuous variables was tested using the skewness-kurtosis test [27]. None of these were normally distributed; therefore, these were summarised as median and interquartile range (IQR).

Threshold analyses were used to identify optimum thresholds for CVD risk, overall and by subtype, and SWB. Logistic regression models were fitted for each ethnic group to predict the binary outcome of CVD or CVD subtype based on the aforementioned predictors. Second, to determine the optimal threshold for a continuous predictor, receiver operating characteristic (ROC) analysis was used using the ‘pROC’ package in RStudio v4.1.0 [28]. The threshold which maximised Youden’s index [29], which provides a balance between sensitivity and specificity, was identified. The robustness of these findings was assessed through K-fold cross-validation (*K* = 5, 10, 15, 20, and 25) and sensitivity analyses to ensure the generalisability and reliability of the identified thresholds.

Adjusted predicted incidence of CVD and CVD subtypes, and linear predictions of SWB, was calculated for each ethnicity. Odds ratios and mean differences of the comparison between Whites and ethnic minorities were calculated at important BMI and WC values.

In primary analyses, cases with missing data were excluded to maintain the integrity of the main investigation. To assess the impact of missingness, sensitivity analyses were conducted using multiple imputation to generate several plausible datasets with imputed values for the variables with missing values. The ‘mice’ package in R was utilised for multiple imputation, and chained equations were employed to impute missing values based on other variables in the dataset. Analyses were then conducted using the imputed values to explore the influence of missing data on the primary analysis results. Then, analyses were conducted using the imputed values to explore the influence of missing data on the primary analysis results. Statistical significance was set at 5%.

## Results

### Summary Characteristics

A total of 296,767 UKB participants were included in the study. The median age of these participants was 57 years, and there were more females in this sample than males. All ethnic groups had median BMI in the overweight category except for East Asians with a BMI of 23.7 kg/m^2^. Median WC ranged between 86 and 92 cm in all ethnic groups except East Asians who had a median WC of 79 cm. Of the 136,696 participants diagnosed with CVD in this study, 94% were White, 0.5% were Mixed, 2.5% were South Asian, 1.9% were Black, 0.3% were East Asian and 0.9% were other. The remaining 0.4% had missing ethnicity in the dataset (Table 1). Median SWB was 75 out of 100 across all ethnicities, indicating that participants were moderately to very happy. White participants had the highest median wellbeing score of 75 (IQR = 16.7), then East Asians (score = 73.3; IQR = 12.5), then the remaining four ethnic groups all with a score of 70.8 and an IQR of 13.3 (Mixed ethnicity) or 12.5 (South Asian, Black, Other).

**Table 1:** Baseline demographic characteristics of the analysable population overall and split by ethnic group

	Overall (*N* = 296,767)		White (*N* = 279,011)		Mixed (*N* = 1189)		South Asian (*N* = 6894)		Black (*N* = 5220)		East Asian (*N* = 928)		Other (*N* = 2825)	
Variable	Med/N	IQR/%	Med/N	IQR/%	Med/N	IQR/%	Med/N	IQR/%	Med/N	IQR/%	Med/N	IQR/%	Med/N	IQR/%
Age	57	13	57	13	50	13	53	14	51	13	53	12	53	14
Body mass index	26.47	5.65	26.44	5.61	26.26	6.00	26.46	5.18	28.70	6.53	23.68	4.67	26.99	6.08
Waist circumference	89	18	89	19	86	18	91	15	92	16	79	16	89	18
Sex														
Female	164,332	55.2%	153,997	55.2%	1,196	63.3%	3,252	47.2%	3,086	59.1%	602	64.9%	1,665	58.9%
Male	133,609	44.8%	125,014	44.8%	693	36.7%	3,642	52.8%	2,134	40.9%	326	35.1%	1,160	41.1%
Smoking status														
Never	168,596	56.8%	155,558	52.4%	974	51.7%	5352	78.4%	3669	70.8%	738	79.8%	1748	62.2%
Previous	101,765	34.3%	98,173	33.1%	615	32.6%	883	12.9%	912	17.6%	126	13.6%	708	25.2%
Current	26,549	8.9%	24,500	8.3%	295	15.7%	591	8.7%	604	11.6%	61	6.6%	354	12.6%
Depression in last 6 m														
No	125,214	86.3%	121,194	86.4%	597	79.5%	1,145	86.3%	902	84.9%	294	88.3%	672	82.7%
Yes	19,872	13.7%	19,115	13.6%	154	20.5%	182	13.7%	161	15.1%	39	11.7%	141	17.3%
Current alcohol intake														
Never	21,936	7.4%	16,651	6.0%	195	10.3%	2,906	42.3%	1,045	20.1%	241	26.0%	731	26.0%
Special occasion	32,253	10.8%	27,847	10.0%	351	18.6%	1,308	19.1%	1,486	28.6%	333	35.9%	779	27.7%
1–3 times a month	33,195	11.2%	31,059	11.1%	273	14.5%	559	8.1%	745	14.3%	115	12.4%	310	11.0%
1–2 times a week	75,783	25.5%	72,441	26.0%	436	23.1%	985	14.4%	1,060	20.4%	115	12.4%	504	17.9%
3–4 times a week	71,646	24.1%	69,653	25.0%	343	18.2%	610	8.9%	517	9.9%	55	5.9%	269	9.6%
Daily	62886	21.1%	61254	22.0%	290	15.4%	496	7.2%	350	6.7%	69	7.4%	218	7.8%
Moderate PA days														
0	34,746	12.2%	32,554	12.1%	224	12.6%	827	13.7%	538	11.3%	126	14.6%	332	13.1%
1	24,150	8.5%	22,800	8.5%	141	7.9%	473	7.8%	398	8.4%	81	9.4%	185	7.3%
2	43,479	15.2%	41,003	15.3%	271	15.2%	867	14.4%	714	15.1%	110	12.7%	387	15.2%
3	44,109	15.5%	41,561	15.5%	273	15.3%	882	14.6%	755	15.9%	144	16.7%	365	14.4%
4	28,923	10.1%	27,321	10.2%	160	9.0%	529	8.8%	535	11.3%	56	6.5%	239	9.4%
5	42,119	14.8%	39,327	14.7%	283	15.9%	1,023	17.0%	798	16.8%	120	13.9%	430	16.9%
6	15,434	5.4%	14,349	5.3%	112	6.3%	357	5.9%	341	7.2%	56	6.5%	154	6.1%
7	52,402	18.4%	49,524	18.4%	317	17.8%	1,069	17.7%	663	14.0%	170	19.7%	450	17.7%
Dominant handgrip	30	16	30	16	30	14	26	14	30	16	26	14	28	15
CVD (any)														
No	136,696	45.9%	128,237	46.0%	1,008	53.4%	2,917	42.3%	2,130	40.8%	513	55.3%	1,362	48.2%
Yes	161,245	54.1%	150,774	54.0%	881	46.6%	3,977	57.7%	3,090	59.2%	415	44.7%	1,463	51.8%

*CVD* cardiovascular disease, *IQR* interquartile range, *Med* median, *N* number, *PA* physical activity

### Threshold Analysis: Fine-Tuning Predictions for Precision Public Health

Threshold analysis assessed the predictive accuracy of CVD and SWB models. Using a logistic regression model for the CVD outcome, all but one of the covariates was statistically significant at the 5% level, moderate physical activity. Compared to the White ethnic group, South Asians and Blacks had significantly higher odds of CVD, 21% and 31% increased odds, respectively. Furthermore, higher age, BMI, WC, previous or current smokers, having depression in the past 6 months and males had higher odds of CVD. Increased handgrip strength and more frequent alcohol intake were protective of CVD.

All variables were statistically significant in the linear regression model that was fit to SWB. All non-White ethnic groups were significantly associated to lower SWB compared to Whites, with the Black group associated with a three-point decrease. Greater age, BMI, physical activity, handgrip and alcohol intake increased SWB. The remaining significantly decreased wellbeing. Full results are presented in Table 2.

**Table 2:** Model estimates for CVD and subjective wellbeing using the significant associations from the path model structure

	Cardiovascular disease		Subjective wellbeing	
Model estimates	OR (95% CI)	RSE	Estimate (95% CI)	RSE
Ethnicity				
White (reference)	0.00		0.00	
Mixed	1.04 (0.89, 1.21)	0.08	− 1.46*** (− 2.23, − 0.69)	0.39
South Asian	1.21** (1.08, 1.36)	0.07	− 1.08*** (− 1.66, − 0.50)	0.30
Black	1.31*** (1.16, 1.49)	0.08	− 3.14*** (− 3.82, − 2.46)	0.35
East Asian	1.19 (0.96, 1.47)	0.13	− 1.63** (− 2.74, − 0.51)	0.57
Other	0.92 (0.79, 1.06)	0.07	− 1.73*** (− 2.50, − 0.97)	0.39
Age	1.08*** (1.08, 1.08)	0.001	0.12*** (0.11, 0.13)	0.004
Body mass index	1.07*** (1.06, 1.07)	0.003	0.03** (0.01, 0.06)	0.01
Waist circumference	1.01*** (1.01, 1.02)	0.001	− 0.07*** (− 0.08, − 0.06)	0.005
Moderate PA days	1.00 (1.00, 1.00)	0.002	0.21*** (0.19, 0.23)	0.01
Dominant handgrip	0.99*** (0.99, 1.00)	0.001	0.06*** (0.05, 0.06)	0.004
Smoking status				
Never (reference)	0.00		0.00	
Previous	1.13*** (1.11, 1.16)	0.01	− 0.71*** (− 0.82, − 0.60)	0.06
Current	1.21*** (1.16, 1.27)	0.03	− 2.34*** (− 2.56, − 2.12)	1.11
Alcohol intake				
Never (reference)	0.00		0.00	
Special occasions only	0.98 (0.92, 1.04)	0.03	− 0.41* (− 0.72, − 0.10)	0.16
1–3 times a month	0.88*** (0.83, 0.93)	0.03	0.07 (− 0.23, 0.37)	0.15
1–2 times a week	0.87*** (0.82, 0.91)	0.02	0.95*** (0.68, 1.22)	0.14
3–4 times a week	0.84*** (0.80, 0.89)	0.02	1.25*** (0.98, 1.52)	0.14
Daily	0.89*** (0.85, 0.94)	0.02	0.92*** (0.64, 1.19)	0.14
Depression in 6 m				
No (reference)	0.00		0.00	
Yes	1.38*** (1.33, 1.42)	0.02	− 7.87*** (− 8.04, − 7.70)	0.09
Sex				
Female (reference)	0.00		0.00	
Male	1.33*** (1.28, 1.38)	0.03	− 0.32*** (− 0.50, − 0.13)	0.10
CVD				
No (reference)			0.00	
Yes			− 1.00*** (− 1.11, − 0.89)	0.06
Constant	0.001*** (0.001, 0.001)	0.0001	72.86*** (72.19, 73.53)	0.34

*CI* confidence interval, *CVD* cardiovascular disease, *OR* odds ratio, *PA* physical activity, *RSE* robust standard error

*P < 0.05; **P < 0.01; ***P < 0.001

The predictive ability of the logistic regression model was tested using the estimated optimum cutoff point, and corresponding values for area under the receiver operating characteristic curve (AUROC), sensitivity and specificity were calculated to assess its performance, the results of which are presented in Table 3. The probability threshold at which the logistic model best classifies someone as having CVD for the overall sample is 0.52, with differing thresholds for individual ethnicities between 0.52 and 0.60. The model captures 62 to 65% of true positive cases and 64 to 68% of true negative cases depending on ethnic group, which translates to an AUROC of 64 to 65%, suggesting the model has moderate discrimination ability between CVD statuses.

**Table 3:** The predictive ability of the logistic regression model of CVD; overall and by ethnicity

Accuracy	All ethnicities	White	Mixed	South Asian	Black	East Asian	Other
CVD							
Probability threshold	0.52	0.52	0.52	0.59	0.60	0.66	0.54
Sensitivity	0.64	0.64	0.64	0.62	0.65	0.62	0.62
Specificity	0.67	0.66	0.64	0.68	0.65	0.66	0.68
AUROC	0.65	0.65	0.64	0.65	0.65	0.64	0.65

The *F*-test gave a *p*-value less than 0.001, suggesting that the model is statistically significant. *R*-squared value was 10.52% which indicates that this model explains 10.52% of the variability of SWB. Both SWB and the linear predictor of the model were split into two groups using a range of cut-offs to identify the optimum cut-off, such that those scoring above this cut-off are assigned the value 1 to signify high SWB and those below assigned 0 to signify low SWB. The cut-off which maximises Youden’s cut-off was the predictive value of 75, which gives a sensitivity of 84% and a specificity of 35%, giving an AUROC of 63%.

#### Cardiovascular Subtypes

The predictive performance of these models was assessed. The area under the curve was between 0.55 and 0.77, sensitivity was between 0.51 and 0.68, and specificity was between 0.56 and 0.68, suggesting moderate predictive accuracy. Results are presented in Appendix Table 1.

### Ethnic-Specific Incidence Rate of CVD at BMI and WC Cutoffs

**Fig. 1:**
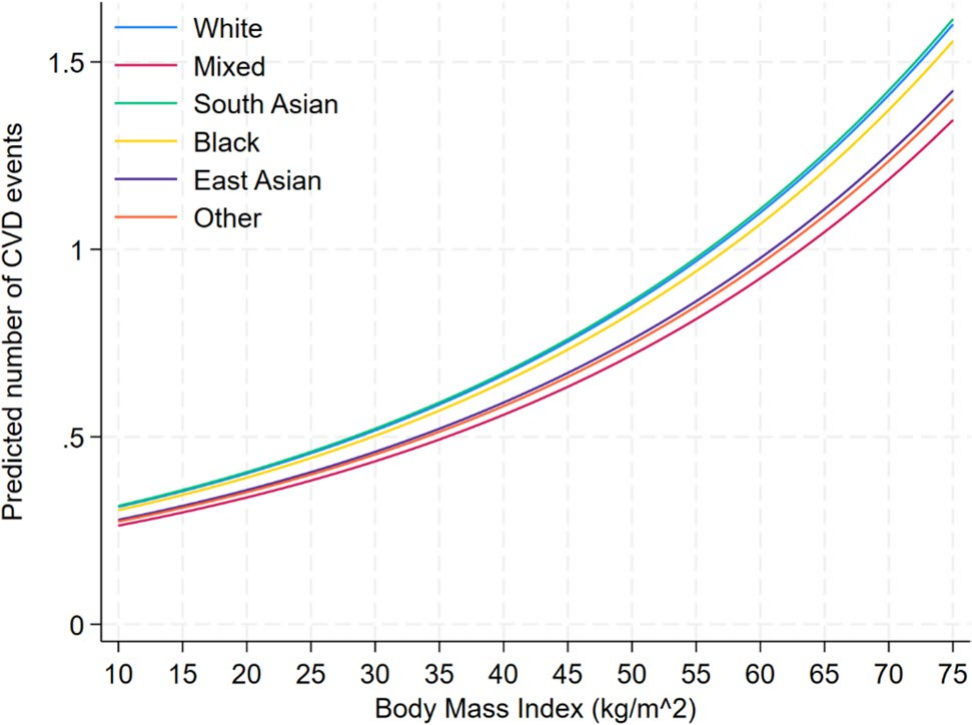
Predicted number of CVD events based on BMI for each ethnic group

**Fig. 2:**
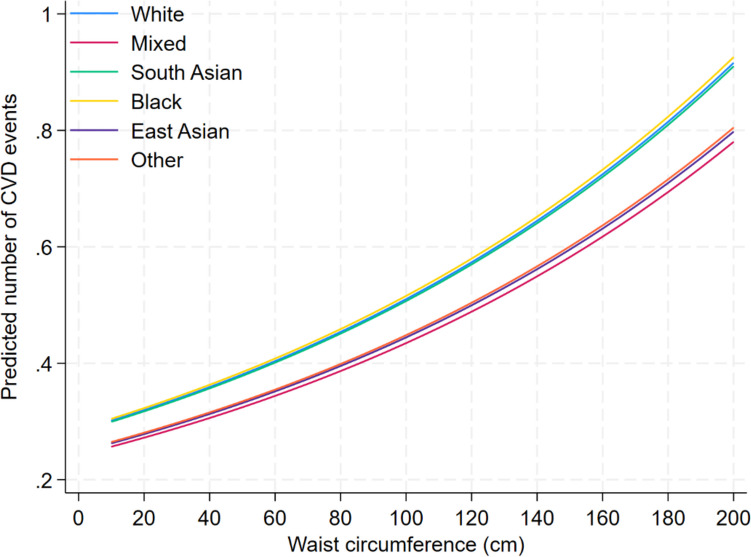
Predicted number of CVD events based on WC for each ethnic group

Participants of mixed or other ethnicity had significantly lower CVD rates compared to Whites across all BMI and WC cutoffs, indicated by the omission of OR = 1 (Table 4). However, all ethnic minority groups had significantly worse predicted SWB scores irrespective of BMI or WC cutoff. Figures 1 and 2 present the predicted number of CVD events split by ethnicity for a range of BMI and WC values. In these figures, there is a clear grouping of ethnicity who is at a higher risk of CVD and who is at a lower risk, with Whites, South Asians and Blacks.

**Table 4:** Odds ratios and mean difference comparisons of predicted CVD events and SWB

		Cardiovascular disease events			Subjective wellbeing		
	Ethnic group	OR	95% CI		MD	95% CI	
Body mass index (kg/m^2^) groups							
18	White	Ref	Ref	Ref	Ref	Ref	Ref
	Mixed	0.941	0.908	0.975	− 2.763	− 3.542	− 1.985
	South Asian	1.003	0.973	1.035	− 2.023	− 2.643	− 1.404
	Black	0.989	0.957	1.022	− 4.398	− 5.093	− 3.703
	East Asian	0.959	0.907	1.013	− 1.803	− 2.921	− 0.686
	Other	0.953	0.920	0.988	− 2.710	− 3.469	− 1.952
25	White	Ref	Ref	Ref	Ref	Ref	Ref
	Mixed	0.930	0.893	0.969	− 2.763	− 3.487	− 2.039
	South Asian	1.004	0.970	1.040	− 2.023	− 2.577	− 1.470
	Black	0.987	0.952	1.024	− 4.398	− 5.018	− 3.778
	East Asian	0.951	0.890	1.016	− 1.803	− 2.895	− 0.712
	Other	0.945	0.907	0.984	− 2.710	− 3.414	− 2.007
30	White	Ref	Ref	Ref	Ref	Ref	Ref
	Mixed	0.921	0.879	0.965	− 2.763	− 3.493	− 2.033
	South Asian	1.005	0.965	1.045	− 2.023	− 2.588	− 1.459
	Black	0.986	0.946	1.027	− 4.398	− 5.016	− 3.780
	East Asian	0.944	0.876	1.018	− 1.803	− 2.907	− 0.700
	Other	0.938	0.895	0.982	− 2.710	− 3.421	− 2.000
35	White	Ref	Ref	Ref	Ref	Ref	Ref
	Mixed	0.911	0.863	0.961	− 2.763	− 3.537	− 1.990
	South Asian	1.005	0.959	1.054	− 2.023	− 2.645	− 1.402
	Black	0.984	0.938	1.032	− 4.398	− 5.058	− 3.738
	East Asian	0.937	0.860	1.022	− 1.803	− 2.944	− 0.663
	Other	0.930	0.881	0.981	− 2.710	− 3.466	− 1.955
Waist circumference (cm) groups							
80	White	Ref	Ref	Ref	Ref	Ref	Ref
	Mixed	0.935	0.897	0.974	− 2.905	− 3.633	− 2.178
	South Asian	0.997	0.963	1.032	− 1.991	− 2.554	− 1.429
	Black	1.005	0.967	1.044	− 4.158	− 4.783	− 3.534
	East Asian	0.943	0.885	1.006	− 2.556	− 3.649	− 1.463
	Other	0.946	0.909	0.986	− 2.810	− 3.518	− 2.102
90	White	Ref	Ref	Ref	Ref	Ref	Ref
	Mixed	0.931	0.891	0.973	− 2.905	− 3.628	− 2.183
	South Asian	0.997	0.962	1.034	− 1.991	− 2.543	− 1.439
	Black	1.005	0.966	1.046	− 4.158	− 4.771	− 3.546
	East Asian	0.940	0.878	1.006	− 2.556	− 3.652	− 1.461
	Other	0.943	0.904	0.985	− 2.810	− 3.512	− 2.108
100	White	Ref	Ref	Ref	Ref	Ref	Ref
	Mixed	0.927	0.885	0.972	− 2.905	− 3.647	− 2.163
	South Asian	0.997	0.959	1.036	− 1.991	− 2.564	− 1.419
	Black	1.006	0.964	1.049	− 4.158	− 4.787	− 3.530
	East Asian	0.936	0.870	1.007	− 2.556	− 3.670	− 1.442
	Other	0.940	0.898	0.984	− 2.810	− 3.531	− 2.089

## Discussion

### General

In this study, we investigated the relationship between important demographic characteristics, CVD, and SWB in a large cohort of UKB participants. Our findings revealed notable associations between demographic factors and both CVD and SWB across different ethnic groups. Threshold analysis was applied to determine the optimal predictive performance of the models constructed. As the study delved into threshold analysis, it became evident that optimal cutoff points differed among ethnic groups, shedding light on nuanced patterns within CVD and CVD subtypes. Furthermore, this study provided CVD risk at key BMI and WC values for each ethnic group and compared this to the prevailing White ethnicity of the UK, uncovering distinct patterns of adjusted-risk stratification. Specifically, our analysis revealed two distinct groupings of ethnicities, with Whites, Blacks, and South Asians exhibiting higher risk profiles for CVD compared to East Asians, mixed and other ethnic groups.

The modelling process demonstrated a moderate predictive ability with a sensitivity of 0.64, a specificity of 0.67 and an AUROC of 0.65 using the optimum cut-off of *p* = 0.52, which suggests that the model has a reasonable ability to discriminate between individuals with and without CVD across all ethnicities. While these metrics emphasise the model’s reasonable ability, it is important to acknowledge the evolving nature of cardiovascular risk assessment. The field has advanced through the exploration of novel biomarkers and lifestyle variables, but a persistent challenge emerges as a substantial number of myocardial infarction cases are still classified as low risk by existing methods and models [30]. Although promising biomarkers like B-type natriuretic peptides show notable predictive capacity, their integration raises concerns about exacerbating social disparities in CVD outcomes [31].

Notably, variations in predictive performance were observed among ethnic groups. The South Asian, Black and East Asian groups exhibited higher optimum cut-offs, suggesting the variables in the models affect cardiovascular health differently in various ethnic groups, necessitating ethnic-specific risk thresholds. Sensitivity and specificity metrics across all ethnicities demonstrate a balanced performance of the model in correctly identifying both positive and negative cases of CVD. The sensitivity values range from 0.62 to 0.65, while specificity ranges from 0.64 to 0.68. The AUROC values, ranging from 0.64 to 0.65 for different ethnic groups, indicate a moderate level of discrimination and are within a broadly acceptable range [32], though the subtle variations may indicate nuanced differences in the model’s performance for specific populations.

When modelling SWB, higher age, BMI, physical activity, handgrip strength and frequent alcohol intake increased SWB. Increased WC, smoking, depression, males and CVD all decreased SWB. As with the CVD model, better physical health increased wellbeing, and poorer habits such as smoking decreased wellbeing. Regarding the predictive model of SWB, the optimum cut-off is the predicted score of 75 out of 100 with its associated sensitivity of 0.84 and specificity of 0.35. The identified cut-off value of 75 may have practical implications, such as in screening programs; however, Youden’s index attempts to maximise the balance between sensitivity and specificity, such a large disparity between the two means future research should consider the implications of this trade-off in the context of subjective wellbeing assessment and potentially explore alternative cut-off values tailored to specific ethnicities.

### Strengths and Limitations

This study benefits from a large and diverse cohort sources from the UKB, offering ample data to explore associations between demographic factors, CVD risk and SWB across various ethnic groups. However, the cross-sectional nature of the study poses limitations, restricting the ability to establish causal relationships between demographic factors, CVD risk and SWB, instead establishing correlations. Although the UK Biobank is a longitudinal dataset, a cross-sectional approach was selected to efficiently examine baseline associations in this study, thereby maximising sample size. Additionally, at the time of analysis, sufficient follow-up data for specific variables of interested was either unavailable or not yet fully curated.

Given that the UKB started data collection between 2006 and 2010, the environmental, societal and health contexts in the UK may have shifted, especially in the wake of major events like the COVID-19 pandemic. As suck, the well-being outcomes presented may not fully reflect the current realities in 2024.

The synergies between the incorporation of past analysis and the threshold analyses showcase a comprehensive approach to the understanding of the research question and strengthen the robustness of the findings. Each analysis brings a unique perspective to the intricate relationship between the variables, building upon the preceding analysis and creating a more complete narrative. Furthermore, feature importance analysis serves as a powerful tool to discern the predictors that exert the most influence on the outcomes of interest. The limited explanatory power of the models for non-White ethnic groups should be acknowledged. With *R*-squared values between 20 and 30%, this indicates that there are factors which are unaccounted for that the current model does not capture.

Moreover, reliance on self-reported measures for variables such as smoking status, alcohol intake and depression introduces potential biases, impacting the accuracy of results. Exclusion of cases with missing data may further limit the generalisability of findings, despite efforts to assess the impact of missingness through sensitivity analyses. While the UK Biobank cohort offers diversity, findings may not fully extend to populations outside the UK or to ethnic groups not represented in the dataset, or the population outside of the ‘well’ population which volunteered for participation in the UKB. Additionally, while logistic regression models demonstrated moderate predictive ability, the AUROC values indicated only moderate discrimination, suggesting the need for additional variables to enhance predictive accuracy.

While this study benefits from the large sample size and extensive data available through the UKB, it is important to note that the cohort is not fully representative of the general UK population. The UKB tends to suffer from ‘healthy volunteer’ bias, as participants to be healthier, older and of higher socio-economic status compared to the wider population, which may introduce selection bias and limit the generalisability of our findings. Additionally, population weights and strata were not included in the statistical analysis, as UKB does not provide population weights, and analyses are usually conducted without such adjustments. This limitation should be considered when interpreting the results, as they may not fully reflect broader population-level trends. Future studies should aim to replicate these findings in more representative samples to validate the associations observed here.

### Implications and Future Research

The findings of this study have potential clinical implications, particularly in the assessment and management of CVD and SWB. Healthcare professionals can use the identified demographic factors, such as age, BMI and lifestyle behaviours, to inform risk stratification and tailor interventions aimed at reducing CVD risk and improving SWB. Understanding the associations between demographic factors, CVD risk and SWB can inform public health strategies aimed at promoting cardiovascular health and mental wellbeing in diverse populations. Targeted interventions addressing modifiable risk factors, such as smoking cessation programs or lifestyle modification initiatives, may help reduce health disparities and improve overall population health.

Future research should prioritise longitudinal studies to establish causal relationships over time. By collecting data at multiple time points, these studies can elucidate the temporal sequence of events and identify critical periods for intervention. Further exploration of additional variables, such as genetic predisposition, is warranted to enhance our understanding of the complex relationship between ethnicity, demographic factors, CVD and SWB. Randomised controlled trials evaluating lifestyle interventions, psychosocial support programs and community-based initiatives can provide valuable evidence for clinical and public health practice. Furthermore, external validation of predictive models developed in this study, and other studies through a systematic review, using independent cohorts is necessary to assess their generalisability and reliability across different populations, ensuring the applicability of findings in diverse settings.

## Conclusion

In conclusion, our study provides evidence to consider ethnic-specific BMI ranges for normal, underweight and overweight categories, as well as healthy waist circumference limits, for CVD risk assessment. The identification of ethnic-specific risk thresholds in predictive models highlight the importance of tailored approaches to risk assessment and intervention. Further research is warranted to compare and validate these findings in other populations and settings, ensuring their applicability beyond the UK Biobank cohort.

## Supplementary Information

Below is the link to the electronic supplementary material.

Supplementary file1 (DOCX 31 KB)

## Data Availability

Researchers can access the data through an established application process which is found at the following website: https://www.ukbiobank.ac.uk/enable-your-research/apply-for-access.
