# Supplementary material for: Ethnic-Specific Threshold Analysis and BMI and Waist Circumference Cutoffs for Cardiovascular Disease and Subjective Wellbeing: Results using Data from the UK Biobank
Source: J Racial Ethn Health Disparities. 2024 Oct 11;12(6):3968–78. doi: 10.1007/s40615-024-02193-9 (PMC12644207; doi:10.1007/s40615-024-02193-9)
Supplement: Supplementary file 1 — Supplementary file1 (DOCX 31 KB) [file 40615_2024_2193_MOESM1_ESM.docx]

Appendix Table 1. Results of threshold analyses of cardiovascular subtypes

|  | **Acute rheumatic fever** | **Chronic rheumatic heart disease** | **Hypertensive diseases** | **Ischaemic heart diseases** | **Pulmonary heart diseases** | **Other forms of heart diseases** | **Cerebrovascular diseases** | **Diseases of arteries, arterioles and capillaries** | **Diseases of veins, lymphatic vessels and lymph nodes** | **Other and unspecified** |
| --- | --- | --- | --- | --- | --- | --- | --- | --- | --- | --- |
| **Model estimates** | | | | | | | | | | |
| Ethnicity |  |  |  |  |  |  |  |  |  |  |
| White (ref) |  |  | 0.00 | 0.00 | 0.00 |  |  |  |  |  |
| Mixed |  |  | 1.12 | 1.21 | 1.06 |  |  |  |  |  |
| South Asian |  |  | 1.58*** | 2.08*** | 0.28** |  |  |  |  |  |
| Black |  |  | 1.75*** | 0.74* | 1.45 |  |  |  |  |  |
| East Asian |  |  | 1.70*** | 0.79 | 0.65 |  |  |  |  |  |
| Other |  |  | 1.18 | 0.99 | 0.80 |  |  |  |  |  |
| Age | 1.08*** | 1.10*** | 1.08*** | 1.09*** | 1.05*** | 1.08*** | 1.08*** | 1.04*** | 1.03*** | 1.07*** |
| Body mass index | 1.02** | 1.05*** | 1.09*** | 1.05*** | 1.03*** | 1.02*** | 1.03*** | 0.97*** | 1.02*** |  |
| Waist circumference |  |  | 1.02*** | 1.01*** | 1.02*** | 1.01*** |  | 1.01** | 1.01*** | 1.01*** |
| Moderate PA days |  |  | 0.99*** |  |  |  |  | 0.01* | 1.02*** |  |
| Dominant handgrip |  | 0.99** | 0.99*** | 0.99*** |  |  | 0.99*** | 0.99*** |  | 0.99*** |
| Smoking status |  |  |  |  |  |  |  |  |  |  |
| Never (ref) |  |  | 0.00 | 0.00 | 0.00 | 0.00 | 0.00 | 0.00 | 0.00 | 0.00 |
| Previous |  |  | 1.10*** | 1.26*** | 1.08 | 1.09*** | 1.21*** | 1.30*** | 1.08*** | 1.18*** |
| Current |  |  | 1.12*** | 1.51*** | 1.38*** | 1.13*** | 1.48*** | 1.91*** | 1.13*** | 1.45*** |
| Alcohol intake |  |  |  |  |  |  |  |  |  |  |
| Never (ref) |  | 0.00 | 0.00 | 0.00 | 0.00 | 0.00 | 0.00 | 0.00 | 0.00 | 0.00 |
| Special occasions only |  | 0.95 | 1.00 | 0.96 | 0.92 | 0.90* | 0.88 | 0.89 | 0.97 | 0.83* |
| 1-3 times a month |  | 0.81 | 0.90** | 0.85** | 0.85 | 0.82*** | 0.79** | 0.79** | 0.95 | 0.77*** |
| 1-2 times a week |  | 0.82* | 0.91** | 0.80*** | 0.74*** | 0.79*** | 0.72*** | 0.76*** | 0.91* | 0.69*** |
| 3-4 times a week |  | 0.81* | 0.91** | 0.75*** | 0.65*** | 0.80*** | 0.64*** | 0.75*** | 0.87*** | 0.61*** |
| Daily |  | 0.75** | 0.98 | 0.67*** | 0.67*** | 0.80*** | 0.74*** | 0.78*** | 0.86*** | 0.61*** |
| Depression in 6 m |  |  |  |  |  |  |  |  |  |  |
| No (ref) |  | 0.00 | 0.00 | 0.00 | 0.00 | 0.00 | 0.00 | 0.00 | 0.00 | 0.00 |
| Yes |  | 1.25*** | 1.27*** | 1.46*** | 1.31*** | 1.27*** | 1.48*** | 1.37*** | 1.29*** | 1.60*** |
| Sex |  |  |  |  |  |  |  |  |  |  |
| Female (ref) | 0.00 | 0.00 | 0.00 | 0.00 |  | 0.00 | 0.00 | 0.00 | 0.00 | 0 |
| Male | 0.81** | 1.67*** | 1.39*** | 2.81*** |  | 1.52*** | 1.91*** | 1.37*** | 0.92*** | 1.62*** |
| Constant*** | 0.00003 | 0.00001 | 0.0001 | 0.0001 | 0.0001 | 0.0005 | 0.0002 | 0.004 | 0.01 | 0.0004 |
| **Accuracy** | | | | | | | | | | |
| Probability threshold | 0.003 | 0.015 | 0.333 | 0.090 | 0.021 | 0.147 | 0.031 | 0.041 | 0.134 | 0.027 |
| AUROC | 0.73 | 0.71 | 0.68 | 0.68 | 0.57 | 0.62 | 0.71 | 0.58 | 0.56 | 0.68 |
| Sensitivity | 0.51 | 0.61 | 0.66 | 0.68 | 0.67 | 0.65 | 0.58 | 0.61 | 0.56 | 0.56 |
| Specificity | 0.62 | 0.66 | 0.67 | 0.68 | 0.62 | 0.64 | 0.65 | 0.60 | 0.56 | 0.62 |
